# Glaukos iStent^®^ Trabecular Micro-Bypass

**DOI:** 10.4103/0974-9233.56227

**Published:** 2009

**Authors:** Louis D. Nichamin

**Affiliations:** From the Laurel Eye Clinic, 50 Waterford Pike, Brookville, PA 15825

**Keywords:** Intraocular pressure, glaucoma, iStent

## Abstract

The iStent trabecular micro-bypass system (Glaukos Corp. Laguna Hills, CA) was developed to address the limitations of current medical and surgical therapies for glaucoma treatment. The iStent^®^ is inserted *ab interno* through a small temporal clear corneal incision, bypassing the trabecular meshwork and placed in Schlemm's canal at the lower nasal quadrant. Implantation of this stent into Schlemm's canal allows aqueous humor to drain directly from the anterior chamber into Schlemm's canal bypassing the obstructed trabecular meshwork. For this review, a Medline search was performed using the terms “trabecular micro-bypass stent” and “trabecular bypass stent.” The online abstract database for the American Academy of Ophthalmology was also reviewed. Abstracts which duplicated published articles were excluded. All relevant papers (n is equal to three) and abstracts (n is equal to one) were included in this review. Multiple, prospective multi-country, clinical trials have demonstrated the safety and efficacy of iStent in reducing IOP, when compared to traditional treatment modalities, while reducing/ eliminating the need for ocular antihypertensive drugs when implanted in OAG patients during combined cataract surgery or in patients with glaucoma refractory to traditional treatment modalities.

## INTRODUCTION

Glaucoma poses a significant clinical and financial burden on the world healthcare community affecting more than 60.5 million people by the year 2010 and increasing to nearly 80 million cases by 2020.^1^ Of these patients, 74% will have primary open-angle glaucoma (POAG), the number two cause of blindness worldwide. The rapidly increasing incidence of POAG has created a significant demand for cost-effective clinical solutions that address the physiological, clinical, and patient management challenges of the disease. The iStent^®^ trabecular micro-bypass device was developed by Glaukos^®^ to address many of these challenges and the limitations of current prescription and surgical therapies.

## ISTENT^®^ DESIGN

**Figure 1:**
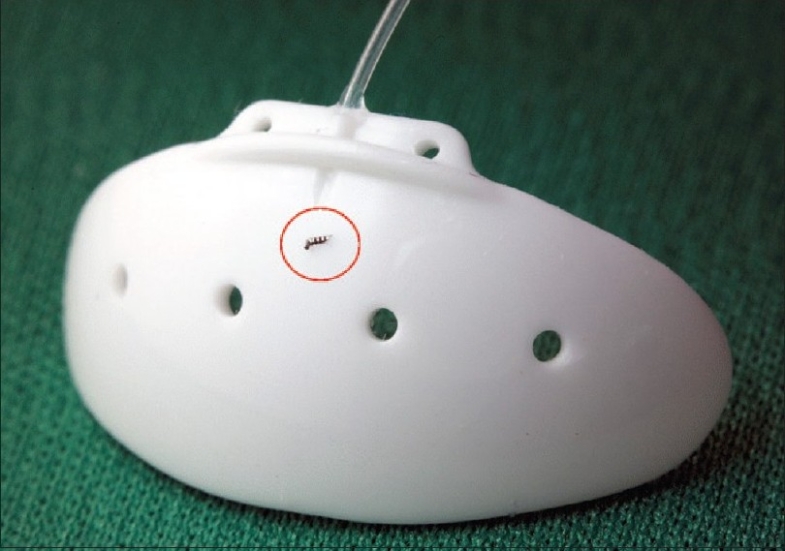
iStent^®^ is the smallest device ever implanted in a Human eye. (image: iStent^®^ on the Baerveldt implant)

The iStent^®^ trabecular micro-bypass is a heparin-coated, nonferromagnetic, surgical grade titanium stent less than one mm in length and approximately 0.3 mm in height, 1/5000 of the size of the Baerveldt implant [Figure 1].

The iStent^®^ is inserted *ab interno* through a small temporal clear corneal incision, bypassing the trabecular meshwork, and placed in Schlemm's canal at the lower nasal quadrant. The dimensions of the stent are customized for a natural fit and retention within the 270*μ* canal space, with three retention arches to ensure secure placement.

## MECHANISM OF ACTION

**Figure 2:**
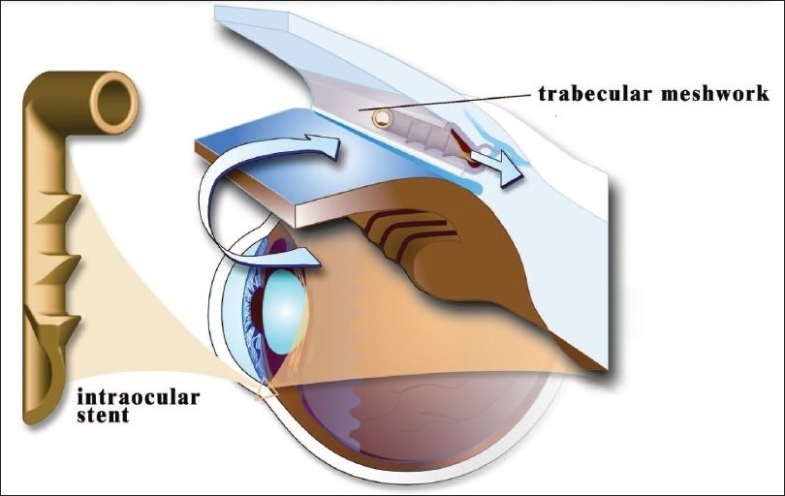
iStent^®^ restores physiologic outflow of Aqueous Humor

The primary cause of ocular hypertension (OHT) in patients with POAG is abnormality of the trabecular meshwork, with up to 75% of resistance to outflow found in the juxtacanicular tissue.^2^ By creating a patent bypass through Schlemm's canal, the iStent^®^ re-establishes physiologic outflow and significantly reduces intraocular pressure (IOP).^3^ *In vitro* data has demonstrated that placement of one iStent^®^ can improve facility of outflow by 84% (p<.003)^3^ [Figure 2].

## CLINICAL RESULTS

Multiple, prospective, multi-country, clinical trials have demonstrated that the iStent^®^ safely and effectively reduces IOP while reducing or eliminating the need for ocular antihypertensive drugs when implanted in patients with OAG in combined cataract surgery.^4^–^6^

In a 24-month, uncontrolled, multicenter, multi-country evaluation of 58 patients with uncontrolled open-angle glaucoma (including pseudoexfoliation and pigmentary) and cataract, the iStent^®^ demonstrated superior statistically and clinically significant results in reducing IOP and medication use versus cataract surgery alone.^4^ Patients underwent clear cornea phacoemulsification followed by *ab interno* gonioscopically guided implant of the iStent^®^. After implantation of the device, patients were more likely to achieve target IOP with fewer ocular antihypertensive medications than prior to implantation. At baseline, mean IOP was 21.7 mm Hg ± 3.98 and was reduced to 17.4 mm Hg ± 2.99 at month 12. Notably, after implantation of iStent^®^; 62% of patients achieved an IOP of 18 mm Hg, 26% had an IOP of 15 mmHg, and 69% achieved an IOP of 21 mmHg without the use any ocular antihypertensive medications. Overall, the iStent^®^ significantly reduced the mean number of medications needed to control IOP at one year by 1.2 fewer medications (*p*<.001). There were no reported incidences of hypotony, postsurgical flat chambers and/or choroidal effusion, and nor was significant reduction of visual acuity noted at 12 months.^4^

## PATIENT SELECTION

The iStent^®^ significantly reduces IOP in patients with early-to-moderate OAG, pigmentary or pseudoexfoliative glaucoma in combination with cataract surgery. The implant of the device allows for future treatment options as it does not interrupt the natural infrastructure of the eye. The iStent^®^ is a sensible clinical option for patients with OAG who find it habitually or financially difficult to adhere to their prescription glaucoma regimen.

## SURGICAL TECHNIQUE

The iStent^®^ is preloaded in a single-use, light release force, sterile applicator with a secure, rotatable grip to facilitate manipulation and placement into Schlemm's canal. It is then delivered in a disposable, sealed sterile tray. Two orientations of the stent are available for the right (OD) and left eye (OS).

The iStent^®^ is implanted through the same small, temporal, clear corneal incision used for phacoemulsification or a 1.5 mm incision when the stent is implanted as a stand-alone procedure. Unlike more invasive, filtering procedures, iStent^®^ implantation can be performed under topical anesthesia, without the formation of a filtering bleb. The procedure is quite safe with minimal to no risk of hypotony due to the physiologic preservation of the trabecular meshwork aiming to ensure the natural episcleral back pressure of 8 - 11 mm Hg.^6^

**Figure 3:**
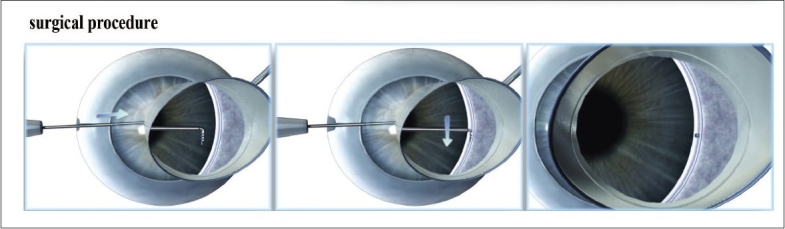
Surgical Technique

Prior to implanting the iStent^®^, the angle anatomy and targeted stent site must be in clear view. For best possible angle visualization; iStent^®^ insertion should be performed from the temporal side with the microscope magnified 12X and tilted towards the surgeon. The patient's head is tilted away from the surgeon [Figure 3].

**Figure 4:**
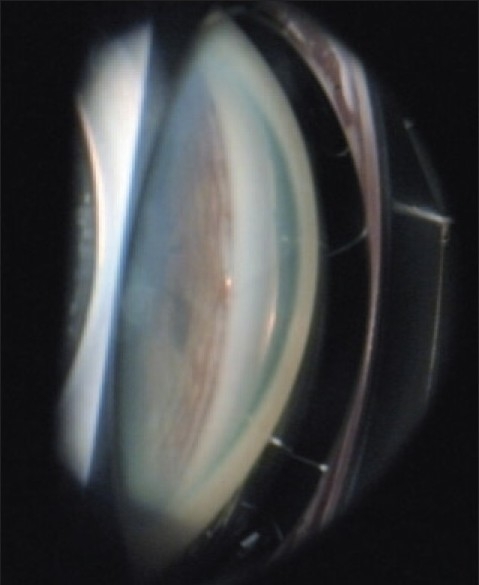
iStent^®^ implanted in Schlemm's Canal

The tip of the stent should approach the trabecular meshwork at 15° angle to facilitate penetration of the tissue. Excessive resistance indicates that the approach is too perpendicular to the trabeculum. Once the stent is covered with meshwork it is released by pressing the applicator button. Only the proximal end of the stent remains visible in the anterior chamber. The iStent^®^ is seated into position by gently tapping the side of the snorkel with the applicator tip. It should be parallel to the iris plane with the inner part covered by the meshwork and the lumen protruding from Schlemm's canal. A small reflux of blood from the Schlemm's canal reflects correct positioning of the stent [Figure 4].

Extraction of the viscoelastic material and hydration of the corneal incision conclude the procedure. Standard postoperative medication should be used as determined by the surgeon.

## DISCUSSION

The iStent^®^ trabecular micro-bypass device addresses many of the clinical, patient management challenges of glaucoma. The iStent^®^ will allow surgeons to intervene earlier in glaucoma management to achieve target IOP, reduce medication use and slow progressive optic neuropathy while avoiding the risks associated with more complex surgical interventions. Data demonstrates that implantation of the iStent^®^ device is safe and provides sustained and significant reduction in IOP over a 12-month period. The iStent^®^ provides a sustainable foundation in re establishing physiologic outflow, achieving target intraocular pressures, and reducing/eliminating the need for ocular antihypertensive medication.
